# Self-Assembly and Multifaceted Bioactivity of a Silver(I) Quinolinate Coordination Polymer

**DOI:** 10.1021/acs.inorgchem.1c02110

**Published:** 2021-09-21

**Authors:** Sabina W. Jaros, Agnieszka Krogul-Sobczak, Barbara Bażanów, Magdalena Florek, Dominik Poradowski, Dmytro S. Nesterov, Urszula Śliwińska-Hill, Alexander M. Kirillov, Piotr Smoleński

**Affiliations:** † Faculty of Chemistry, University of Wroclaw, F. Joliot-Curie 14, 50-383 Wrocław, Poland; ‡ Faculty of Chemistry, University of Warsaw, Pasteura 1, 02-093 Warsaw, Poland; § Department of Pathology, Wrocław University of Environmental and Life Sciences, Norwida 31, 50-375 Wrocław, Poland; ∥ Department of Biostructure and Animal Physiology, Wrocław University of Environmental and Life Sciences, Kożuchowska 1, 51-631 Wrocław, Poland; ⊥ Centro de Química Estrutural and Departamento de Engenharia Química, Instituto Superior Técnico, Universidade de Lisboa, Av. Rovisco Pais, 1049-001 Lisbon, Portugal; # Department of Analytical Chemistry, Faculty of Pharmacy, Wroclaw Medical University, Borowska 211 A, 50-566 Wrocław, Poland; % Research Institute of Chemistry, Peoples’ Friendship University of Russia (RUDN University), 6 Miklukho-Maklaya st., 117198 Moscow, Russia

## Abstract

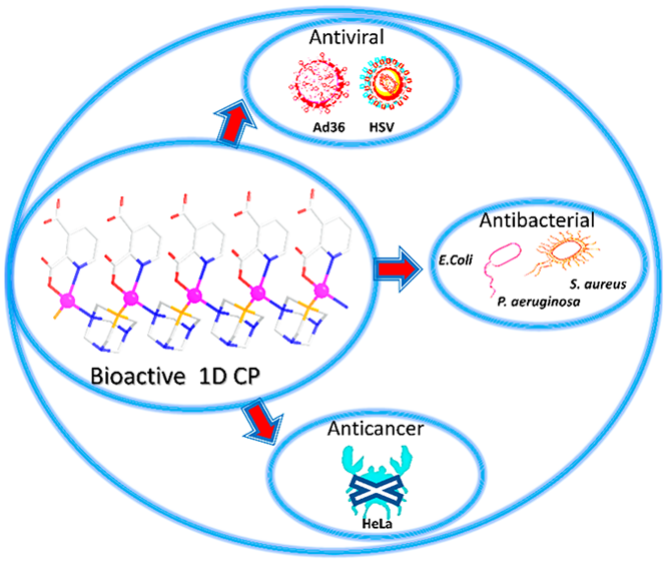

Coordination polymers have emerged as a new class of potent biologically active agents due to a variety of important characteristics such as the presence of bioactive metal centers and linkers, low toxicity, stability, tailorable structures, and bioavailability. The research on intermediate metabolites has also been explored with implications toward the development of selective anticancer, antimicrobial, and antiviral therapeutic strategies. In particular, quinolinic acid (H_2_quin) is a recognized metabolite in kynurenine pathway and potent neurotoxic molecule, which has been selected in this study as a bioactive building block for assembling a new silver(I) coordination polymer, [Ag(Hquin)(μ-PTA)]_*n*_·H_2_O (**1**). This product has been prepared from silver oxide, H_2_quin, and 1,3,5-triaza-7-phosphaadamantane (PTA), and fully characterized by standard methods including single-crystal X-ray diffraction. Compound **1** has revealed distinctive bioactive features, namely (i) a remarkable antiviral activity against herpes simplex virus type 1 (HSV-1) and adenovirus 36 (Ad-36), (ii) a significant antibacterial activity against clinically important bacteria (*Staphylococcus aureus*, *Escherichia coli*, and *Pseudomonas aeruginosa*), and (iii) a selective cytotoxicity against HeLa (human cervix carcinoma) cell line. The present work widens a growing family of bioactive coordination polymers with potent antiviral, antibacterial, and antiproliferative activity.

## Abstract 2

A new silver(I) coordination polymer driven by aminophosphine and quinolinate ligands was self-assembled and fully characterized and revealed a potent antiviral, antibacterial, and antiproliferative activity.

## Introduction

Infectious diseases and particularly those of a viral genesis are responsible for igniting most of the global pandemics. Influenza, smallpox, rabies, and Ebola viruses have been harvesting a death toll for centuries and contributed to the deaths of millions of people around the globe.^1−5^ Other infectious agents such as herpes simplex virus (HSV) and human coronaviruses and adenoviruses are ubiquitous and cause numerous diseases, including recurrent keratitis, mild and severe respiratory tract infections, encephalitis, ocular and gastrointestinal tract disorders, and neonatal HSV infections.^1−5^ Despite a huge progress in vaccination, medical technologies, and therapies, treatment of these infections is often just symptomatic without any approved antiviral treatment protocols. Even now, we are defenseless facing the COVID-19 outbreak due to limited antiviral treatment options.^1−3^ Therefore, the identification and development of new antiviral chemicals and treatment approaches is a subject of paramount importance for modern society.

Coordination compounds^6−8^ and especially coordination polymers (CPs)^2,9−12^ built from biocidal metal nodes and/or bioactive organic ligands represent a promising route for developing new metal-based antiviral materials and metallodrugs. Because of some structural and physicochemical fractures, CPs can exhibit an interesting therapeutic outcome with a potential synergic effect of its various components. An assembly of biologically active metal ions and biorelevant organic pillars (metabolic intermediates) into metal–organic network provides an appealing way for designing new bioactive materials.^8−12^

A particularly interesting example of such a biorelevant pillar concerns quinolinic acid (H_2_quin, pyridine-2,3-dicarboxylic acid), which is the main downstream metabolite in the kynurenine pathways in mammals and in aspartate pathways in bacteria and plants.^13^ An appropriate level of this bioligand in cerebrospinal fluids is crucial for correct functioning of the nervous system and preventing the development of multiple sclerosis Huntington’s and Alzheimer’s diseases. On the other hand, perturbations in kynurenine pathways involving a production of H_2_quin and alterations of its concentration are observed in specific neoplastic and viral diseases.^13−21^

In the inflammatory changed cellular microenvironment, quinolinic acid can potentially exert a poisonous effect on the abnormal cancer cells and viruses initiating their programed death. This property of H_2_quin can especially be considered for developing alternative anticancer and antiviral therapies.^14−21^ Moreover, the complexation of quinolinic acid with bioactive metals such as silver can significantly reduce the overall toxicity of H_2_quin on not-perturbated cells, leading to an enhanced bioactivity of the obtained coordination compounds. However, parameters such as poor light and thermal stability, limited shelf life, and/or inadequate solubility and stability in the biological medium (e.g., PBS: phosphate buffered saline) pose obstacles on the development of new silver(I) coordination compounds and their medicinal applications.^22−26^

In contrast to discrete metal complexes, some silver(I)-based coordination polymers represent improved stability and release properties, thus also gaining an impetus for applications in anticancer and antimicrobial treatment.^27−29^ In particular, water-soluble cagelike aminophosphine ligands such as PTA (1,3,5-triaza-7-phosphaadamantane) and its various derivatives constitute an appealing class of linkers for designing bioactive coordination polymers.^30−32^ Notable examples of such silver(I)-based compounds with potent and diverse bioactivity were reported in our previous studies.^33−40^ Within this context and following our major goal in exploring the chemistry and biofunctional applications of silver–PTA coordination polymers, herein we report the self-assembly synthesis, characterization, spectroscopic and structural features, and antiviral, antimicrobial, and antiproliferative properties of a new silver(I) coordination polymer derived from PTA and quinolinic acid, [Ag(Hquin)(μ-PTA)]_*n*_·H_2_O (**1**).

## Results and Discussion

### Synthesis

**Scheme 1:**
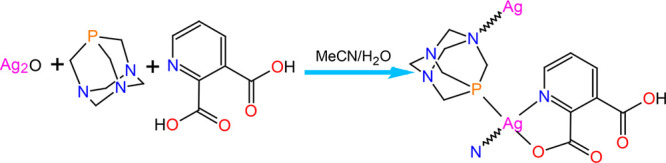
Simplified Representation for the Self-Assembly Synthesis of **1**

A new aqua-soluble silver(I) CP [Ag(Hquin)(μ-PTA)]_*n*_·H_2_O (**1**) was self-assembled by using a mixed-ligand synthetic approach (Scheme 1), namely by reacting silver(I) oxide, quinolinic acid (H_2_quin), and 1,3,5-triaza-7-phosphaadamantane (PTA) in water/methanol medium at ∼25 °C. The product was isolated in a microcrystalline form and characterized by standard methods, including the single-crystal and powder X-ray diffraction analyses. The latter shows a good match between the experimental and simulated patterns, thus confirming a phase purity of the obtained product (Figure S1, Supporting Information).

The IR spectrum of **1** indicates the characteristic ν_as_(COO) and ν_s_(COO) bands of carboxylate groups in the 1700–1365 cm^–1^ region (Figure S2). The Δν value of 229 (Δν refers to a calculated frequency difference between ν_as_ and ν_s_) is in good agreement with the data observed for unidentate carboxylate ligands.^41^ There are also typical ν(C–X) (X = P, N) bands at 1400–500 cm^–1^ associated with the coordinated PTA moieties. The ^1^H NMR spectrum of **1** in D_2_O exhibits a set of signals expected for the aromatic protons of quinolinic acid along with the methylene protons of PTA ligands (Figure S3). The ^31^P{^1^H} NMR spectrum reveals a broad singlet at δ – 75.4 that is consistent with the presence of the coordinated PTA in solution (Figure S4).

Compound **1** is air and light stable in the solid state. It is also stable in the D_2_O solution for at least several days. The solution stability of **1** (25 °C, pD = 6.25, D_2_O) was monitored over time intervals of 0, 1, and 7 days by ^31^P{^1^H} and ^1^H NMR spectroscopy, indicating no appreciable spectral changes under these conditions (Figures S3–S6). However, **1** is less stable in DMSO-*d*_6_ and slowly decomposes, as attested by an evolving PTA=O peak in the ^31^P{^1^H}NMR spectrum after 1 day (Figure S7). The observed molar conductivities of **1** measured in H_2_O and DMSO solutions are 14.35 and 2.07 mS m^2^ mol^–1^, respectively, and thereby indicate its electrolyte nature.

The ESI-MS data (Figures S8 and S9) reveled the presence in solution of two characteristic cationic species, namely [Ag(PTA)_2_]^+^ and [Ag_2_(PTA)_2_(Hquin)]^+^. Both fragments exhibit high intensity and expected isotopic distribution. On the basis of these observations, we can conclude that the 1D polymeric chain structure of **1** is maintained in solid state, while in solution the compound dissociates into stable lower mass fragments. This is also in agreement with the data observed for other reported [Ag(PTA)(carboxylate)]_*n*_ coordination polymers.^37,38^

### Crystal Structure

**Figure 1:**
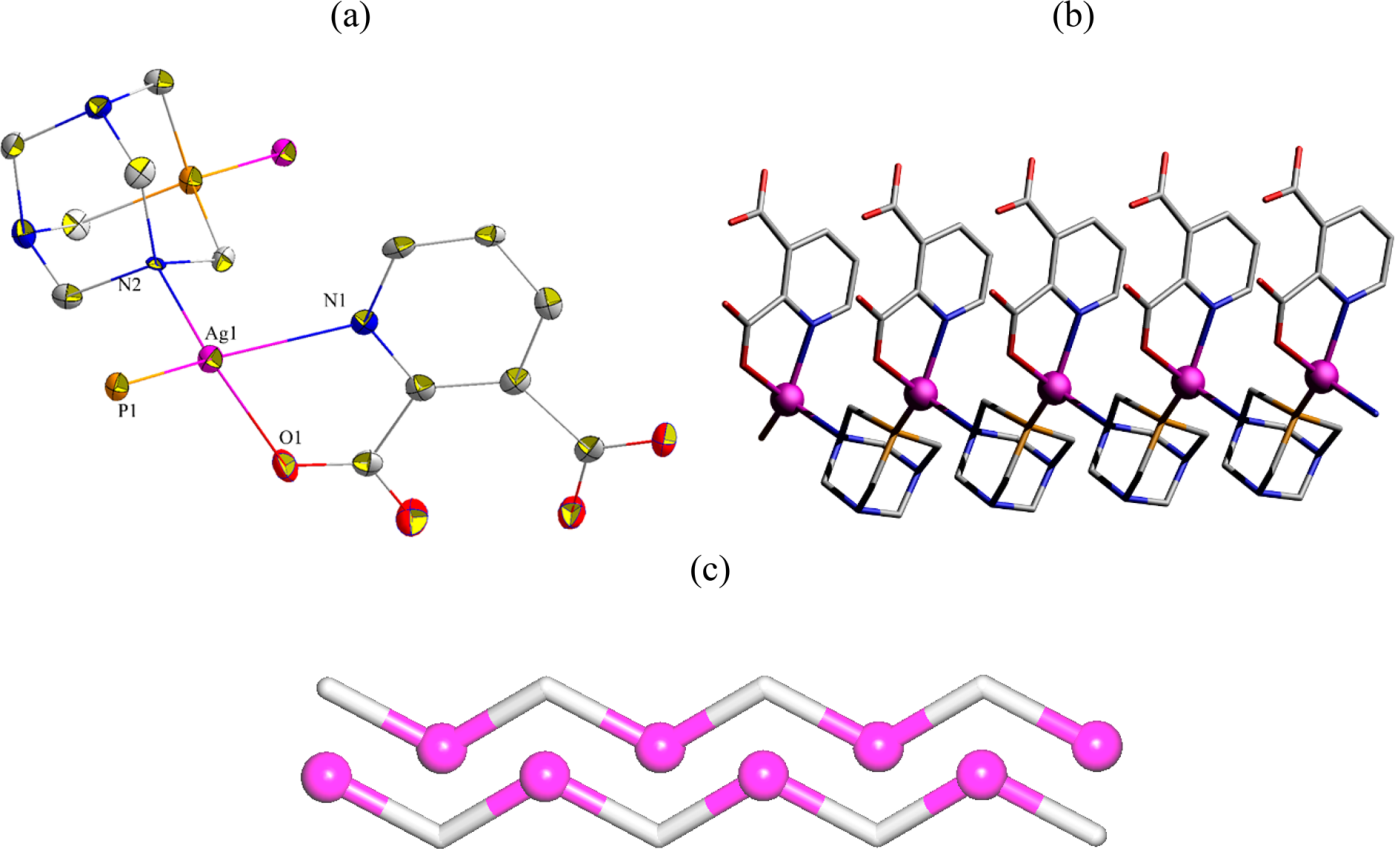
Crystal structure of **1**. (a) Coordination environment around silver(I) center and ligands connectivity. (b) 1D coordination polymer chain. (c) Topological representation of two adjacent chains of a 2C1 type. (a, b) H atoms and crystallization H_2_O were omitted; Ag (magenta), N (blue), P (orange), O (red), C (gray). (c) Ag centers (magenta balls), centroids of μ-PTA linkers (gray).

The structure of **1** comprises a silver(I) center, a μ-PTA linker, a terminal Hquin^–^ ligand, and a crystallization water molecule (Figure 1a). The Ag1 center is four-coordinate and is bound by the N and P donors from two μ-PTA moieties and a pair of N and O atoms from the Hquin^–^ ligand, resulting in a distorted tetrahedral {AgPN_2_O} environment. The μ-PTA linkers combine two Ag1 centers into a linear 1D coordination polymer chain with a shortest Ag···Ag separation of 6.8252(3) Å that is equal to the *b* unit cell parameter (Figure 1b). From a topological perspective, such a chain can be classified within a 2C1 type (Figure 1c). The Ag–N [2.358(6)–2.382(5) Å], Ag–P [2.3332(18) Å], and Ag–O [2.360(5) Å] bond distances are in typical range for Ag–PTA–carboxylate CPs.^35,37,38^

### Antiviral Properties

Both compounds **1** and H_2_quin lower the enveloped HSV virus titer by 6.25 and 5.50 log, respectively (Table 1), indicating a total viral killing effect. This effect is up to 2 orders of magnitude higher if compared to acyclovir (4 log reduction, equivalent to ≥99.99% inactivation), which is a common drug to treat herpes. In the case of nonenveloped Ad-36 virus, a decrease of 4 log in virus titer was observed when using **1** and H_2_quin. Given the absence of officially registered drugs against adenoviruses, [Co(NH_3_)_6_]Cl_3_ was selected as a positive control on account of being the most promising compound in the therapy of adenoviral infections.^4^ However, under the influence of [Co(NH_3_)_6_]Cl_3_, the virus level decreases only by 3 log (99.9% reduction). Although quinolinic acid is a neurotoxin because of its highly virucidal properties, it seems appropriate to consider the use of H_2_quin to treat viral infections.

**Table 1:** Virus Titer Reduction Expressed on a Logarithmic Scale after Treatment with Various Antiviral Compounds

virus	compd **1**	H_2_quin	acyclovir	[Co(NH_3_)_6_]Cl_3_
HSV	6.25	5.5	4	nd^a^
Ad36	4	4	nd^a^	3

a Not detectable virus titer reduction (nd).

It is also interesting to note that a combination of silver ions with H_2_quin in **1** results in even superior activity against HSV. Because **1** can inactivate the enveloped herpes virus, it may also be effective against other enveloped viruses, such as orthomyxoviridae (human and animal influenza viruses), coronaviridae (including SARS-CoV), flaviviridae, and poxviridae as well as blood-borne viruses including HBV, HCV, and HIV. Moreover, the virucidal efficacy of **1** against the nonenveloped adenovirus, which is more resistant to disinfection than the enveloped viruses, makes this compound promising as a potential drug or disinfectant.

Hence, an approach to treat viral infections with H_2_quin derivatives is completely innovative. So far, the role of quinolinate phoshoribosyltransferase (QPRT), an enzyme that catalyzes the formation of nicotinate mononucleotide from 5-phosphoribosyl 1-pyrophosphate (PRPP) and quinolinic acid, has been considered.^42^ Wang et al. described QPRT as an antiviral host factor against virus infection in the course of hepatitis C.^43^ H_2_quin along with 3-HK (3-hydroxyanthranilic acid), 3-HAA (3-hydroxyanthranilic acid), or KYNA (kynurenic acid) are intermediate products of the kynurenine pathway. Interestingly, the concentration of H_2_quin increases with sustained activation of the immune system. However, no clear explanation for this correlation has been found so far.^44^ It is undeniable that higher levels of H_2_quin appear as a result of a viral infection, and at the same time, we have proven *in vitro* that H_2_quin is virucidal. This may indicate that an increased concentration of H_2_quin is a form of defense against the pathogen.

### Antibacterial Activity

Antibacterial properties of **1** were evaluated by using strains of three species representing clinically important bacteria, namely *Staphylococcus aureus*, *Escherichia coli*, and *Pseudomonas aeruginosa*. All the mentioned species were reported as expressing increasing resistance to clinically established antimicrobial therapies,^9,45,46^ though the tested strains were not characterized as resistant or multiresistant. The normalized MIC values obtained for compound **1** (Table 2) suggest its superior antimicrobial activity in comparison to AgNO_3_ that was used as a reference antibacterial. Gram-negative bacteria (*E. coli*, *P. aeruginosa*) revealed a higher susceptibility to **1** if compared to a Gram-negative one (*S. aureus*). However, even in the latter case, the activity of **1** is almost 2-fold superior vs silver(I) nitrate. None of the ligands (H_2_quin, PTA) showed antimicrobial activity at a maximum concentration tested. This is also in agreement with prior data for PTA (MIC > 600 μg mL^–1^)^37^ and H_2_quin (MIC > 1024 μg mL^–1^)^47^ established for the same species of bacteria. The MIC values shown by **1** are comparable to those reported for other Ag-based CPs.^33−40^ On the basis of the aforementioned findings, we can hypothesize that the antimicrobial properties of **1** are mainly associated with the action of Ag^+^ ions, which might be strongly influenced by the type of coordination environment present and binding affinity of O-, N-, and P-donor ligands. However, further studies are required to shed light on the actual mechanism of antimicrobial action of compound **1**.

**Table 2:** Antibacterial Activity of **1**

	MIC [μg mL^–1^]^a^		normalized MIC [nmol mL^–1^]^b^	
strain	compd **1**	AgNO_3_^40^	compd **1**	AgNO_3_^40^
*E. coli*	20	9	45	53
*P. aeruginosa*	20	9	45	53
*S. aureus*	30	20	67	118

a PTA and H_2_quin are not active at maximum tested concentration.

b Values normalized for a molar content of silver in the compounds.

### Cytotoxic Activity

The cytotoxicity effect of compound **1** against NHDF (normal human dermal fibroblasts), A549 (human lung carcinoma), and HeLa (human cervix carcinoma) cell lines was investigated by using MTT assay along with cisplatin and AgNO_3_ as positive controls. After incubation period (72 h), a half-maximal inhibitory concentration (IC_50_) was determined (Table 3). The obtained data revealed a highly selective cytotoxic action of **1** against HeLa cells (IC_50_ = 68.6 ± 7 μM) and its inactivity against human lung carcinoma (A549). The NHDF, used as a normal body tissue model, was significantly resistant when treated with **1**. A lower cytotoxicity of **1** (93.8 ± 10.5 μM) in comparison with cisplatin (16.7 ± 2 μM) for normal body cell line shows that **1** might be a promising compound in anticancer treatment because its cytotoxicity against HeLa is higher than in normal body cell culture and is still lower than the same parameter of cisplatin (Table 3). This observation also proves the selective activity of this compound against various types of neoplasms. The different etiology and pathogenesis of human cervix carcinoma and human lung carcinoma probably will be helpful in explaining this phenomenon (inactivity against A549). A resistance of the latter cell for cisplatin and its potential sensitization ability represent a difficulty for *in vitro* studies,^48^ similarly to human lung carcinoma resistance for chemotherapy.^49,50^

**Table 3:** Cytotoxicity of **1** and Reference Compounds Expressed in Half-Maximal Inhibitory Concentration (IC_50_) Values (μM)

cell line	compd **1**	AgNO_3_^33^	cisplatin^34^	H_2_quin	PTA
NHDF	93.8 ± 10.5	44.2 ± 1.5	16.7 ± 2	nd^a^	nd
A549	nd	176.6 ± 26	33.3 ± 4.2	nd	nd
HeLa	68.6 ± 7.0	176.6 ± 33.2	16.7 ± 3.1	nd	nd

a nd = not detectable.

The lack of blood–brain barrier crossing together with the local administration of **1** can give promising results for further exploration, since there is low toxicity against not only central nervous system cells (quinolinic acid as neurotoxin) but also unaffected body tissues. Moreover, Walczak^44^ suggested that an increase of the quinolinic acid concentration in tissues can be the result of the immune system activation and response against pathogen. Hence, it can be hypothesized that a specific coordination of quinolinate ligand by silver ion plays an important role in the cytotoxicity of **1**.

### Antioxidant Properties

**Figure 2:**
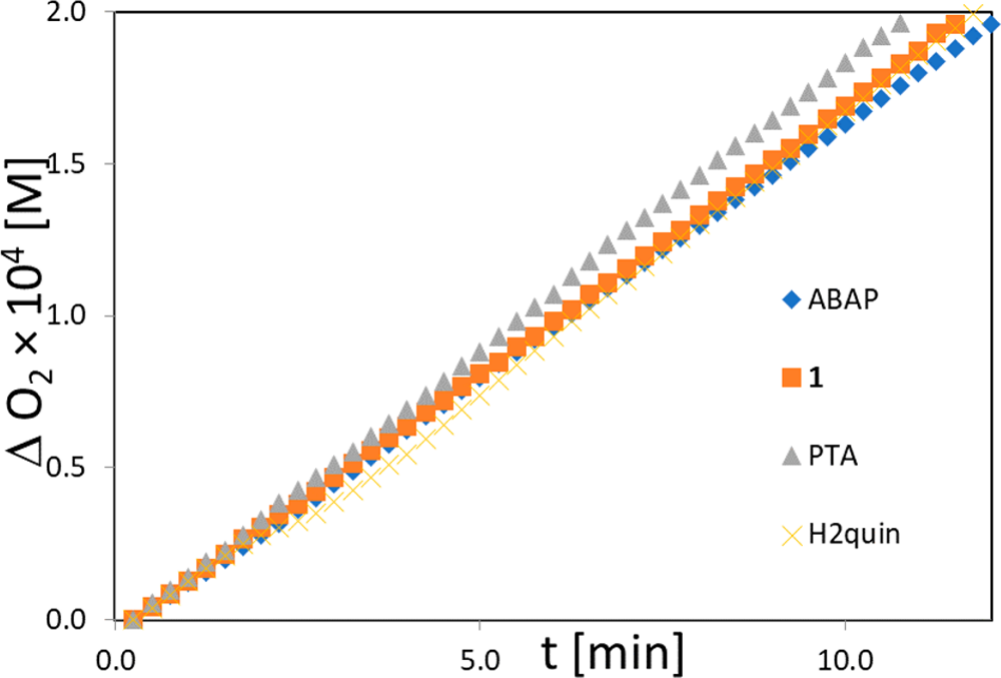
Oxygen uptake for autoxidation of micellar system initiated with ABAP at 37 °C and pH 7.0. Autoxidation in the absence (ABAP, blank) or presence of tested compounds (1 μM): **1**, PTA, or H_2_quin. Final concentration: LinMe 2.74 mM, Triton X-100 8 mM.

The antioxidant behavior of **1** was investigated in the peroxidation of LinMe (methyl linoleate) in the micellar system; plots of oxygen uptake are shown in Figure 2. Autoxidation was initiated by addition of 10 mM water-soluble azo-initiator, 2,2′-azobis(2-amidinopropane) dihydrochloride (ABAP). We monitored the rates of peroxidation in the presence of **1**, PTA, and H_2_quin (Figure 2 and Figures S10–S13). The kinetic plots are substantially the same, which indicates that the tested compounds are kinetically neutral during the peroxidation of micelles initiated with ABAP, thus not exerting antioxidant or prooxidant activity.

### Interaction of **1** with Human Serum Albumin

Human serum albumin (HSA) is one of the most important blood components that plays a main role in the binding and transport of numerous exo- and endogenous ligands such as fatty acids, hormones, toxic metabolites, metals, and drugs. Analysis of the protein interactions with drugs is very important from the pharmacological point of view. To determine the binding parameters between **1** and HSA, mode of action, and structural changes, we used circular dichroism (CD) and fluorescence spectroscopy techniques.

**Figure 3:**
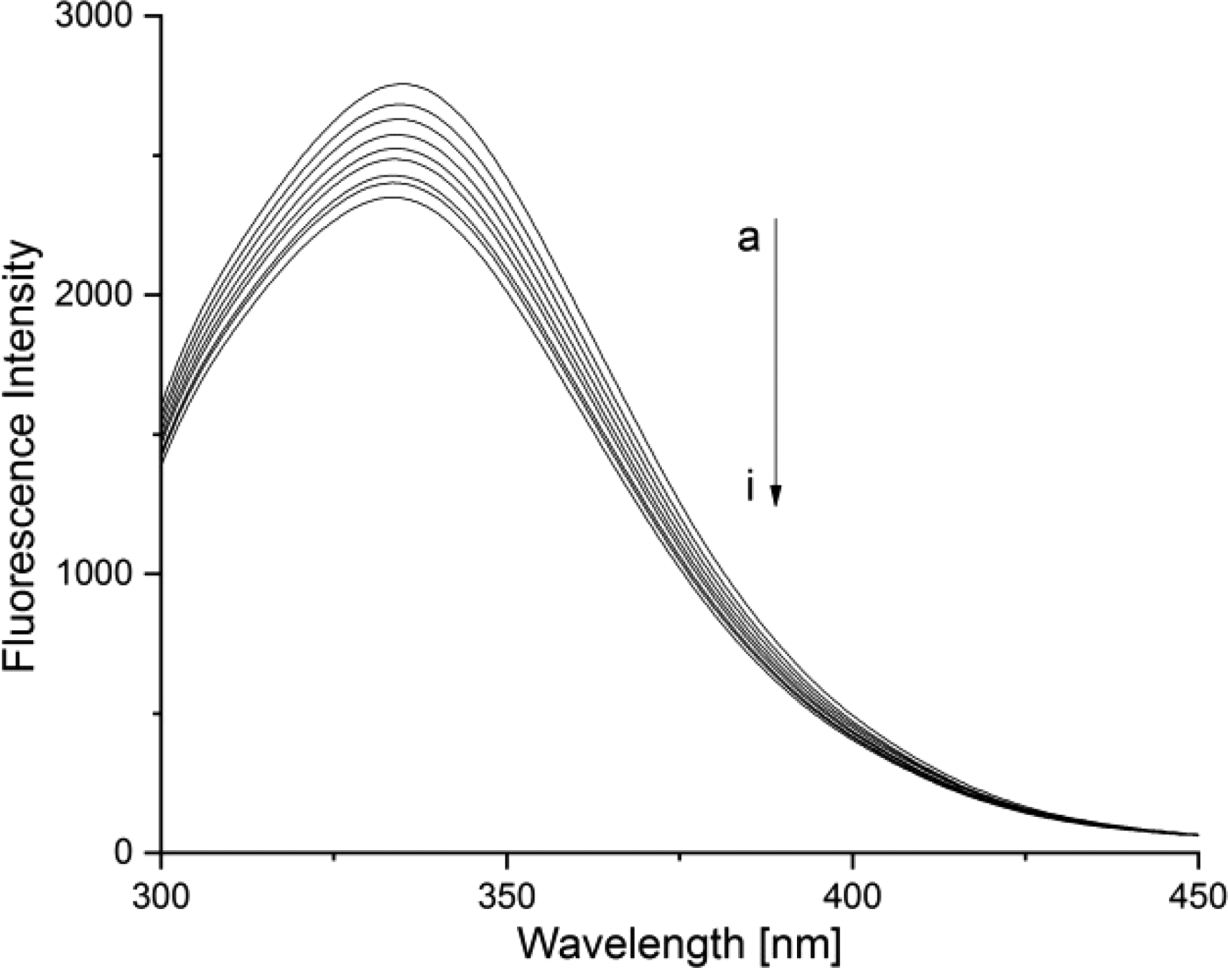
Fluorescence emission spectra in the HSA–**1** system. Lines a–i correspond to the ratios of [HSA]:[Ag] = 1:0–1:16 (λ_ex_ = 280 nm, 310 K, pH 7.40).

The fluorescence emission spectra of the HSA–**1** system are shown in Figure 3. After excitation at 280 nm, human serum albumin reveals a strong fluorescence signal at 334.5 nm. Titration with compound **1** causes a decline of fluorescence signal intensity without a significant shift of the emission peak. This indicates that the interaction between HSA and **1** occurs, and there is no alteration of the microenvironment around the HAS’s chromophore.

**Figure 4:**
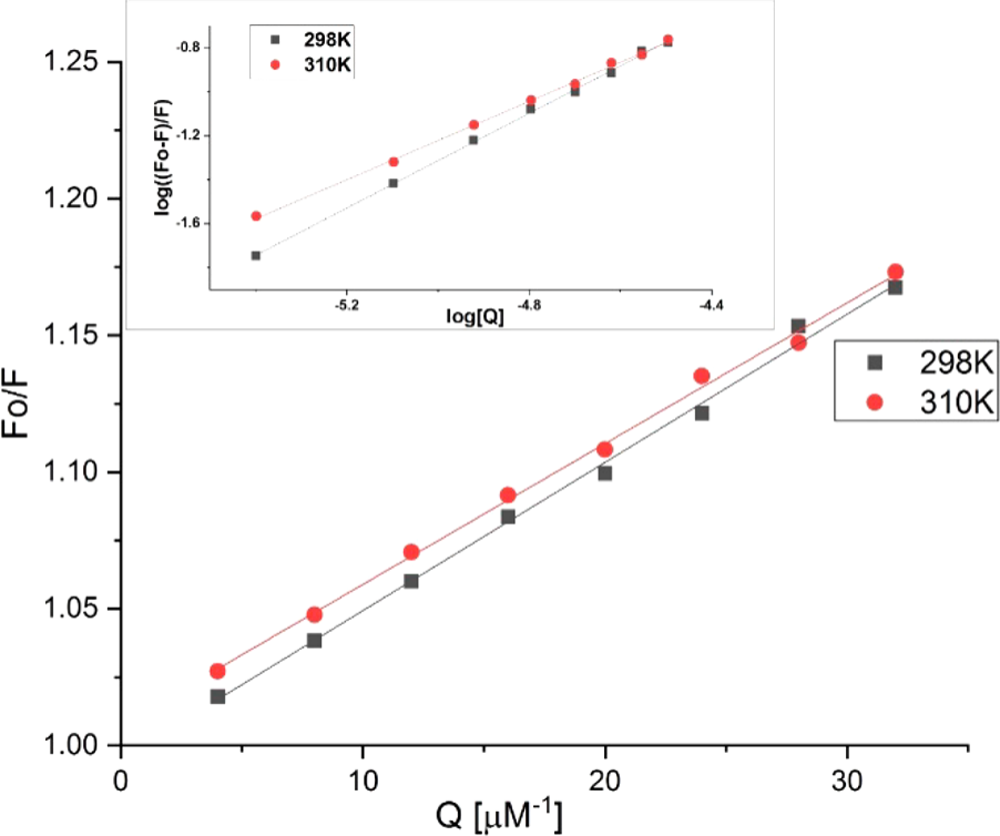
Stern–Volmer plots of the HSA–**1** system at 310 and 298 K (λ_ex_ = 280 nm) (inset: log((*F*_0_ – *F*)/*F*) vs log [Q] plots under the same conditions).

The Stern–Volmer equation is a universal tool to analyze the fluorescence quenching parameters of proteins. On the basis of eqs 1 and 2, we determined the mechanism and fluorescence quenching constants of the HSA–**1** system.
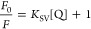
1
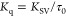
2where *F*_0_ and *F* are the relative fluorescence intensities of protein in the absence and presence of a quencher, respectively, *K*_SV_ is the Stern–Volmer quenching constant, *K*_q_ is the quenching rate constant, [Q] is the quencher concentration, and τ_0_ is the average lifetime of a biomolecule without quencher (for HSA, τ_0_ is 10^–8^ s). The corresponding quenching constants *K*_SV_ for the interaction between HSA and **1** are 5.15 × 10^3^ and 5.42 × 10^3^ M^–1^ at 310 and 298 K, respectively. It is clear that the *K*_SV_ values decrease when temperature increases, and therefore we can conclude a static quenching fluorescence of albumin by **1** (Figure 4). This also reveals the formation of a nonfluorescent complex between **1** and HSA. The reverse effect is generally observed for dynamic quenching processes.

All calculated binding parameters are collected in Table S1. The number of binding sites and association constants between HSA and **1** were calculated based on eq 3.^51^
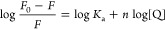
3where *F*_0_ and *F* are the fluorescence intensities in the absence and presence of the quencher, respectively, *K*_a_ is the binding constant, *n* is the number of binding sites, and [Q] is the total concentration of the quencher. The data collected in Table S1 and Figure 4 (inset) show that the binding constant values (*K*_a_) decrease with an increase in temperature when the protein interacts with **1**.

This relationship indicates that **1** forms an unstable complex with the protein, which also undergoes a partial decomposition at higher temperatures. The *N* value approximates to 1, indicating that only one binding site in protein is accessible to compound **1**. Under physiological conditions the binding constant of the system is 1.73 × 10^3^ M^–1^. It points a rather weak affinity of **1** to HSA, which is comparable with the *K*_a_ value of the HSA–cisplatin system (*K*_a_ = 8.52 × 10^2^ M^–1^) and is lower in comparison to other metal complexes.^52−55^

The Ross–Subramanian theory and eqs 4–6 were used to determine the acting forces and thermodynamic parameters in the HSA–**1** system:
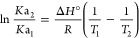
4
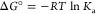
5
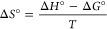
6In these equations, Δ*H*°, Δ*S*°, and Δ*G*° are the enthalpy, entropy, and free energy, respectively, *K*_a_ is the binding constant at the corresponding temperature *T* [K], and *R* is the gas constant.

Usually four types of the binding forces between small molecules and biological macromolecules can be present in the interaction. These include electrostatic forces, hydrophobic and ionic interactions, and van der Waals forces as well as hydrogen bonds. Nevertheless, the contribution of the metal ion in the interaction cannot be excluded in the case of coordination compounds. Moreover, the hydrophobic forces may increase Δ*H*° and Δ*S*°. The hydrogen bonds and van der Waals interactions may decrease them, whereas the electrostatic forces usually make *H*° ≈ 0 and *S*° > 0. In this context, both negative values of Δ*H*° and Δ*S*° indicate that intermolecular interactions play an important role in the binding process of **1** with HSA. Moreover, the negative values of Δ*G*° show that spontaneous processes occur in this system.

We used the circular dichroism technique to determine the effect of **1** on the protein secondary structure. In the intrinsic region (180–250 nm), the CD spectrum of the protein is characterized by two negative bands (Figure S14). A more intense band at 209 nm and a broad and weak band around 221 nm can be assigned to α-helical structure and π → π*/n → π* transitions for the peptide bond, respectively.^56^ Under the experimental conditions, **1** does not generate the CD signal, but as evident from Figure S14, the presence of the silver compound has an effect on the secondary structure of the albumin, causing some changes in the intensity of the protein negative band at 209 nm. The α-helical content of free HSA at 209 nm is equal to 67.23%. A decrease in α-helicity to ∼65.30% was observed after addition of **1** in the molar ratio of [HSA]/[Ag] = 1:10 or 1:20. This indicates that the binding of the ligand with the protein destroys a hydrogen binding network of HSA.

The changes in the protein secondary structure result from the interaction between **1** and HSA. The band intensities of HSA at 209 and 221 nm decreased with the negative Cotton effect through the binding of **1** without causing significant shifts of the peaks, thus clearly indicating that the compound induced a slight decrease in the α-helical structure content of the protein. Reagents concentrations were properly low, and any concentration effect can be excluded. Moreover, no changes in the negative bands at 209 and 221 nm on increasing the concentration of **1** were observed. From these results, it is evident that the effect of **1** on HSA caused secondary structural changes to the protein with a slight loss of the helical stability.

## Conclusions

In summary, we described the self-assembly synthesis as well as structural and biological features of a new silver(I)-based coordination polymer [Ag(Hquin)(μ-PTA)]_*n*_·H_2_O (**1**). By combination of biocidal metal nodes and water-soluble aminophosphine linker with quinolinate(1−) ligand as an important metabolic intermediate, the present compound extends the family of bioactive silver(I) coordination polymers.

A multifaceted antiviral, antibacterial, and cytotoxicity behavior of **1** was investigated in detail, demonstrating its high antimicrobial and antiproliferative potential. In particular, antiviral tests revealed an unprecedented ability of **1** to kill herpes simplex virus type 1 (HSV-1) and adenovirus 36 (Ad-36) with a low toxicity to normal cells. Taking into account the antioxidant measurements, we can conclude that compound **1** is kinetically neutral, while its cytotoxic activity is not a result of its prooxidant properties.

Because compound **1** can inactivate enveloped herpes virus, its efficacy against other enveloped viruses can be envisaged, including such families as orthomyxoviridae (human and animal influenza viruses), coronaviridae, flaviviridae, poxviridae, and blood-borne viruses (e.g., HBV, HCV, and HIV). Moreover, the virucidal efficacy of the CP **1** against the nonenveloped adenovirus, which is more resistant to disinfection than the enveloped viruses, makes this compound promising for consideration as a potential drug or disinfectant. In further research, it would be particularly interesting to investigate a mechanism that would explain the action of **1** as a virucidal agent.

The interaction of **1** with human serum albumin was also investigated by using fluorescence spectroscopy and CD methods. The results prove that the compound interacts with human serum albumin with moderate affinity and forms an unstable complex with the protein. Furthermore, the CD analysis demonstrates that **1** exerts some effect on the protein secondary structure and reduces the α-helix content.

Apart from widening the family of PTA-driven coordination polymers,^30−32^ the present work also provides an important contribution to better understanding of different bioactivity features of such compounds, also revealing their significant potential toward development of novel antiviral and antimicrobial agents. These research lines deserve further development and extension to various combinations of silver centers with other types of carboxylic acid metabolites.

## Experimental Section

### Chemicals

All standard chemicals and solvents were obtained from commercial suppliers. Human serum albumin was acquired from Sigma-Aldrich (A3782, ≥99% purity). The protein stock solution with a concentration of 2 μM was prepared in PBS buffer (pH = 7.40). A stock solution of **1** (833 μM) was prepared by dissolving the compound in demineralized water. Protein concentration was calculated by using ε_HSA_ = 42864 M^–1^ cm^–1^ at 280 nm.^57^ PTA (1,3,5-triaza-7-phosphaadamantane) was synthesized according to a published method.^58,59^

### Analytical Methods

Bruker IFS 1113v (Germany) or BIO-RAD FTS3000MX (BIO-RAD, France) instruments (range 4000–400 cm^–1^) were used to measure the IR spectra (abbreviations: vs, very strong; s, strong; m, medium; w, weak; br, broad). Solution NMR spectra were recorded in D_2_O and DMSO-*d*_6_ by using a Bruker 500/600 AMX spectrometer (Bruker BioSpin MRI GmbH, Germany) at ambient temperature (abbreviations: s, singlet; d, doublet; t, triplet, br, broad). ^1^H chemical shifts (δ) are expressed in ppm relative to Si(Me)_4_, while δ(^31^P) shifts are relative to an external H_3_PO_4_ (85% aqueous solution). ESI-MS(±) spectra were run on a Bruker MicroTOF-Q mass spectrometer with an ESI source by using ∼10^–3^ M solutions of **1** in H_2_O/MeOH. The elemental analyzer Vario ELCube (Elementar Analysen systeme GmbH, Germany) was used for the determination of C, H, and N contents (Laboratory of Elemental Analysis at Faculty of Chemistry, University of Wrocław).

### Synthesis of [Ag(Hquin)(μ-PTA)]_*n*_·H_2_O (**1**)

Silver(I) oxide (0.1 mmol, 23 mg), quinolinic acid (H_2_quin, 0.377 mmol, 63 mg), and PTA (0.2 mmol, 31 mg) were combined in a CH_3_CN/H_2_O (5 mL/5 mL) solution and stirred in air for 1 h to produce a white suspension. This was dissolved by a dropwise addition of an aqueous 1 M solution of NH_3_ (until pH = 8; ∼0.8 mL). The obtained solution was filtered off, and the filtrate was left in a vial to slowly evaporate in air at room temperature. Colorless crystals (including those of X-ray quality) were formed in 1 week. These were then collected and washed with water and methanol and dried in air to give **1** in 40% yield, based on Ag_2_O. Compound **1** is soluble in H_2_O (*S*_25°_ ≈ 0.5 mg mL^–1^) and DMSO (*S*_25°_ ≈ 0.6 mg mL^–1^). Elemental analysis: Calculated for C_13_H_18_AgN_4_O_5_P (MW 449.15): C, 34.76; N, 12.47; H, 4.04. Found: C, 34.96; N, 12.73; H, 3.43; mp 160 °C (dec). Molar conductivity (H_2_O, DMSO, conc = 10^–3^ M): Λ 14.35 and 2.07 mS m^2^ mol^–1^, respectively. IR (KBr, cm^–1^): 3469 (s br) ν(H_2_O + OH), 3071 (w), 2961 (wm) ν_as_(CH), 2923 (m), 1700 (m), 1594 (m) and 1558 (m) ν_as_(COO), 1473 (m), 1454 (m), 1421 (m), 1365 (m) ν_s_(COO), 1301 (m), 1293 (m), 1241 (s), 1163 (m), 1109 (s), 1001 (w), 1038 (w), 1013 (vs), 988 (w), 974 (vs), 958 (m), 948 (s), 898 (w), 853 (w), 807 (s), 795 (s), 751 (s), 735 (w), 699 (w), 643 (m), 605 (vs), 581 (m), 568 (m) 506 (w), 470 (w), 457 (w), 449 (w), 400 (w). ^1^H NMR (500.1 MHz, D_2_O): δ 8.53 (br s, 1H, ^6^H, Hquin), 8.23 (d, 1H, ^4^H, *J*_5_ = 7.6 Hz, Hquin), 7.60 (t, 1H, ^5^H, *J*_4,6_ = 7.6 Hz, Hquin), 4.66 and 4.79 (2d, 6H, *J*_AB_ = 13.0 Hz, NC*H*^A^*H*^B^N, PTA), 4.35 (d, 6H, *J*_P–H_ = 1.9 Hz, PCH_2_N, PTA). ^31^P{^1^H} NMR (202.5 MHz, D_2_O): −75.3 (s, PTA). ESI-MS(±) (H_2_O/MeOH), selected fragments with relative abundance >10%, MS(+), *m*/*z*: 421 (100%) [Ag(PTA)_2_]^+^, 696 (65%) [Ag_2_(PTA)_2_(Hquin)]^+^; MS(−) *m*/*z*: 166 (80%) [Hquin]^−^, 439 (85%) [Ag(Hquin)_2_]^−^, 976 (20%) [Ag_3_(PTA)(quin)(Hquin)_2_]^−^.

### Stability Tests

In this procedure, compound **1** was introduced into a NMR tube and dissolved in 0.6 mL of D_2_O or DMSO-*d*_6_ in air. The ^31^P{^1^H} and ^1^H NMR spectra were monitored for several days at room temperature (Figures S3–S7).

### X-ray Crystallography

Single crystal data collection was performed on a Xcalibur diffractometer (Oxford Diffraction) with a Sapphire2 CCD detector, equipped with an Oxford Cryosystems open-flow nitrogen cryostat, by using ω-scan and a graphite-monochromated Mo Kα (λ = 0.71073 Å) radiation. Cell refinement, data reduction, analysis, and absorption correction were performed with CrysAlis PRO (Rigaku Oxford Diffraction) software.^60^ The structure was solved by direct methods with SHELXT-2014/5 and refined with full-matrix least-squares techniques on *F*^2^ with SHELXL-2018/3.^61,62^ Direct solution in the correct space group *P*2_1_/*n* was unsuccessful. The structure was solved in the space group *P*_*n*_, and then the symmetry was transformed to *P*2_1_/*n* by using the ADDSYM feature of the PLATON program.^63,64^ The H3 atom on the COOH functionality was placed on O3 atom on the basis of the C–O carboxylate distances. The O3–H3 distance was restrained to 0.97 Å. The O–H bond distances in the water molecule O1W were restrained to 0.85 Å and H1···H2 separation to 1.38 Å. All other hydrogen atoms were placed at calculated positions and refined by using the model with *U*_iso_ = 1.2*U*_eq_.

#### Crystal Data for **1**

C_13_H_18_AgN_4_O_5_P, *M* = 449.15, *a* = 13.5460(8) Å, *b* = 6.8252(3) Å, *c* = 17.1564(8) Å, β = 92.148(5)°, *V* = 1585.07(14) Å^3^, *T* = 85(2) K, monoclinic, space group *P*2_1_/*n*, *Z* = 4, Mo Kα, 10636 reflections measured, 3720 independent reflections (*R*_int_ = 0.0778), *R*_1_ = 0.0766 (*I* > 2σ(*I*)), *wR*(*F*^2^) = 0.1408, GoF(*F*^2^) = 1.114. CCDC 2079371 (**1**).

### Cell Cultures

The cell cultures of normal human dermal fibroblasts (NHDF; PromoCell, C-12302, Germany), human lung carcinoma (A549; ATTC, No CCL-185), and human cervix carcinoma (HeLa; ATCC, No CCL-2) were used to evaluate a cytotoxicity of **1**. NHDF and A549 cell lines were cultured in Dulbecco’s Modified Eagle Medium (DMEM, Lonza, Switzerland), while HeLa cells were cultured in Minimum Essential Medium (MEM; Sigma, Germany). The supplementation of 10% fetal bovine serum (FBS, Biological Industries, Kibbutz Beit-Haemek, Israel), 4 mM l-glutamine (Biological Industries, Kibbutz Beit-Haemek, Israel), 100 U/mL of penicillin, and 100 μg/mL of streptomycin (Sigma, Germany) was routinely used in all media.

### Cytotoxicity Assay

The scheme of cytotoxicity assay consisted in enzymatic reduction of soluble tetrazolium salt in metabolically active cells into insoluble purple formazan (MTT test). The concentration of tetrazolium dye was measured colorimetrically by using a spectrophotometric microplate reader (Multiscan Go, Thermo Fisher, USA). The cell cultures were inserted into 96-well plates (Eppendorf, Germany) in a concentration of 105 cells per well. Compound **1** was dissolved in culture medium and diluted to reach the concentration of 100, 50, 20, 10, 5, 2, and 1 μg/mL and then added to cell cultures and incubated in standard conditions (37 °C with a constant flow of 5% CO_2_) for 72 h. After a proper incubation period, the cytotoxicity of **1** was evaluated by using MTT assay (Sigma, Germany). In the MTT procedure, 20 μL of 3-(4,5-dimethylthiazol-2-yl)-2,5-diphenyltetrazolium bromide (1 mg/mL) was added to each well and subsequently incubated for 4 h at 37 °C. The lysis buffer (80 μL) provoked a destruction of cell membranes, which was monitored by spectrophotometric analysis measuring the optical density (OD) of solution. The following formula was used to estimate the viability of investigated cell cultures: cell viability (%) = (average OD for test group/average OD for control group) × 100. The untreated cells were used as a negative control and doxorubicin (a cytostatic routinely used in therapy) was applied as a positive control.^65^

### Antiviral Assay

Antiviral activity tests of **1**, H_2_quin, acyclovir, and [Co(NH_3_)_6_]Cl_3_ were conducted by using human adenovirus 36 (Ad-36 virus-ATCC VR-1610) and herpes simplex virus type 1 (HSV-1-ATCC VR-1493). The above compounds were tested by using EN 14476 [EN 14476 Chemical Disinfectants and Antiseptics—Quantitative Suspension Test for the Evaluation of Virucidal Activity in the Medical Area—Test Method and Requirements (Phase 2/Step 1); European Committee for Standardization: Brussels, Belgium, 2013]. This standard describes a quantitative suspension test for the evaluation of virucidal activity in the medical area (phase 2/step 1), mixing 1 part by volume of test virus suspension (0.1 mL of 1 × 10^8^ TCID_50_ Ad-36 virus or HSV), 1 part by volume of interfering substance (0.1 mL of PBS), and 8 parts by volume of disinfectant (investigated compounds in a concentration of 10 mg/mL). At specified contact times (60 min), aliquots were taken, and serial dilutions up to 10^–8^ of each mixture were prepared. In eight repeats, 50 μL of each dilution was added to the microtiter plate containing a monolayer of confluent A549 or HeLa cells. The plates were observed daily for up to 4 days for the development of viral cytopathic effect by using an inverted microscope (Olympus Corp., Germany; Axio Observer, Carl Zeiss MicroImaging GmbH). Additionally, according to the same procedure, acyclovir (against HSV) or [Co(NH_3_)_6_]Cl_3_ (against Ad36) was tested as a control. Then, residual infectivity was determined. According to PN-EN 14476, a disinfectant is considered as having virucidal effectiveness if within the recommended exposure time the titer is reduced by ≥4 log 10 steps (inactivation ≥99.99%).

### Antibacterial Studies

The antibacterial activity of **1** and ligands (PTA and H_2_quin) was evaluated by using the method of serial dilutions according to Grove and Randall.^66−68^ Two reference strains, *Staphylococcus aureus* PCM 2054 (= ATCC 25923) and *Escherichia coli* PCM 2057 (= ATCC 25922), were obtained from the Polish Collection of Microorganisms of the Institute of Immunology and Experimental Therapy in Wroclaw. A clinical strain of *Pseudomonas aeruginosa* of veterinary origin, identified as previously described, was also used.^37^ Microbial inocula were prepared by using an overnight culture of each strain. These were diluted (1:1000) by using an antibiotic broth (AB). Working dilutions of the tested substances in AB were prepared in 48-well plates to achieve the final concentrations of the substances ([μg mL^–1^]: 60, 50, 40, 30, 20, 10, 9, 8, 7, 6, 5, 4, 3, 2, and 1) after combining 0.9 mL of the working dilution and 0.1 mL of microbial inoculum. Broth sterility and growth controls were performed. The inoculated 48-well plates were incubated at 37 °C for 24 h. The minimum inhibitory concentration (MIC, μg mL^–1^) was defined as the lowest concentration of the compound that fully inhibited the growth of bacteria. For comparison, the MIC values were normalized for the molar content of silver and are represented in a nmol mL^–1^ scale. PTA and H_2_quin show no antimicrobial activity at the maximum concentration tested.

### Preparation of Micelles

Micelles were prepared by using a previously described procedure.^69^ To a glass test tube, 10 μL of methyl linoleate (LinMe) and 5.5 mL of 16 mM Triton X-100 were added and shaken on Vortex for 1 min. Next, 5.5 mL of buffer (pH 7.0) was introduced, and the mixture was shaken again for 1 min. The final concentration of lipid and surfactant in the micellar system was 2.74 mM LinMe and 8 mM Triton X-100. Buffer composition: pH 7.0 phosphate (25 mM KH_2_PO_4_; 14.5 mM NaOH).

### Antioxidant Measurements

The antioxidant behavior of **1** and H_2_quin was measured by monitoring the rate of peroxidation in a heterogeneous model system (micelles). The uptake of dissolved oxygen in micelles during peroxidation was performed at 37 °C by using a Biological Oxygen Monitor equipped with a Clark-type oxygen electrode. To a glass chamber containing a magnetic stirrer, 5 mL of a micellar solution (pH 7.0) was added and then saturated with oxygen. Next, the electrodes were placed inside the chambers, and a peroxidation was initiated by injecting the ABAP solution (final concentration: 10 mM). After 10% of oxygen consumption, 10 μL of the solution of studied compounds was added (final concentration was 1 μM). The influence of tested compounds on the peroxidation was determined graphically. Because of the neutrality of studied compounds (no changes in plots after addition of compounds) to peroxidation of micelles, kinetic parameters were not determined.

### Fluorescence Spectroscopy

Fluorescence measurements were performed by using a Jasco 8200 spectrofluorometer and a 1.0 cm quartz cell. The widths of excitation and emission slits were set at 5.0 nm. The excitation wavelength was set at 280 nm, and the emission was recorded in the 300–500 nm range. Phosphate-buffered saline (PBS, pH = 7.4) was used as a blank. The fluorescence studies were performed by manually titrating 2 mL of HSA (human serum albumin) solution with 10 μL of **1**. The samples were measured after 5 min incubation at 298 and 310 K. The final concentrations of the protein and **1** during titration experiment were calculated by using eqs 7 and 8:
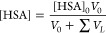
7
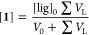
8where [HSA]_0_ and V_0_ are the initial concentration and volume of the protein solution, respectively, the [lig]_0_ is a concentration of stock solution of **1**, and ∑*V*_L_ is total volume of the solution of **1** added during the titration experiment.

### Circular Dichroism Spectroscopy

CD measurements were performed by using a Jasco J-1500 CD spectrometer in a 0.98 mm quartz cuvette. Spectra were recorded after 24 h incubation at 310 K in the 200–250 nm range. A bandwidth of 1 nm, a response time of 2 s, and a scanning speed of 50 nm/min were fixed to obtain final spectra as an average of three scans. The final concentration of the protein was 4 μM, and the molar ratio [HSA]:[**1**] varied from 1:0 to 1:10 and 1:20.

## Supporting Information Available

PXRD patterns (Figure S1), IR, NMR, and MS spectra (Figures S2–S9), separated plots of oxygen uptake (Figures S10–S13), CD spectra of the HSA–**1** system (Figure S14), and Table S1 (PDF)

## Accession Codes

CCDC 2079371 contains the supplementary crystallographic data for this paper. These data can be obtained free of charge via www.ccdc.cam.ac.uk/data_request/cif, or by emailing data_request@ccdc.cam.ac.uk, or by contacting The Cambridge Crystallographic Data Centre, 12 Union Road, Cambridge CB2 1EZ, UK; fax: +44 1223 336033.
