# Supplementary material for: Self-Assembly and Multifaceted Bioactivity of a Silver(I) Quinolinate Coordination Polymer
Source: Inorg Chem. 2021 Sep 21;60(20):15435–44. doi: 10.1021/acs.inorgchem.1c02110 (PMC8527454; doi:10.1021/acs.inorgchem.1c02110)
Supplement: Supplementary file 1 — ic1c02110_si_001.pdf [file ic1c02110_si_001.pdf]

Supporting Information

Self-Assembly and Multifaceted Bioactivity of a Silver(I)-Quinolinate Coordination Polymer

Sabina W. Jaros [†], Agnieszka Krogul-Sobczak [‡], Barbara Bażanów [#], Magdalena Florek [#], Dominik Poradowski [§], Dmytro S. Nesterov [⊥], Urszula Śliwińska-Hill [‖], Alexander M. Kirillov [⊥,^,*] and Piotr Smoleński [†,*]

[†] Faculty of Chemistry, University of Wroclaw, F. Joliot-Curie 14, 50-383 Wrocław, Poland.

[‡] Faculty of Chemistry, University of Warsaw, Pasteura 1, 02-093 Warsaw, Poland.

[#] Department of Pathology, Wrocław University of Environmental and Life Sciences, Norwida 31, 50-375 Wrocław, Poland.

[§] Department of Biostructure and Animal Physiology, Wrocław University of Environmental and Life Sciences, Kożuchowska 1, 51-631 Wrocław, Poland.

[⊥] Centro de Química Estrutural and Departamento de Engenharia Química, Instituto Superior Técnico Universidade de Lisboa, Av. Rovisco Pais, 1049–001 Lisbon, Portugal.

[‖] Department of Analytical Chemistry, Faculty of Pharmacy, Wroclaw Medical University, Borowska 211 A, 50-566 Wrocław, Poland.

[^] Research Institute of Chemistry, Peoples' Friendship University of Russia (RUDN University), 6 Miklukho-Maklaya st., 117198 Moscow, Russia.

Table of Contents

Figure S1. PXRD patterns of compound **1** 3

Figure S2. IR spectrum of **1** 4

Figure S3. ^1H NMR spectrum of **1**(D_2O) 4

Figure S4. $^{31}P\{^1H\}$ NMR spectrum of **1** (D_2O) 4

Figure S5. ^1H NMR spectrum of **1** (D_2O, after one week) 5

Figure S6. $^{31}P\{^1H\}$ NMR spectrum of **1** (D_2O, after one week) 5

Figure S7. $^{31}P\{^1H\}$ NMR spectrum of **1** (DMSO-d_6, after one day) 5

Figure S8. ESI-MS(+) spectrum of **1**. 6

Figure S9. ESI-MS(-) spectrum of **1** 6

Figure S10. Oxygen uptake plot for autoxidation of micelar system without any additive 6

Figure S11. Oxygen uptake plot for autoxidation of micelar system in the presence of **1** 7

Figure S12. Oxygen uptake plot for autoxidation of micelar system in the presence of PTA 7

Figure S13. Oxygen uptake plot for autoxidation of micelar system in the presence of H_2quin 8

Figure S14. CD spectra of the HSA-**1** systems. 8

Table S1. Binding and thermodynamic parameters of the interaction of HSA with **1**. 9

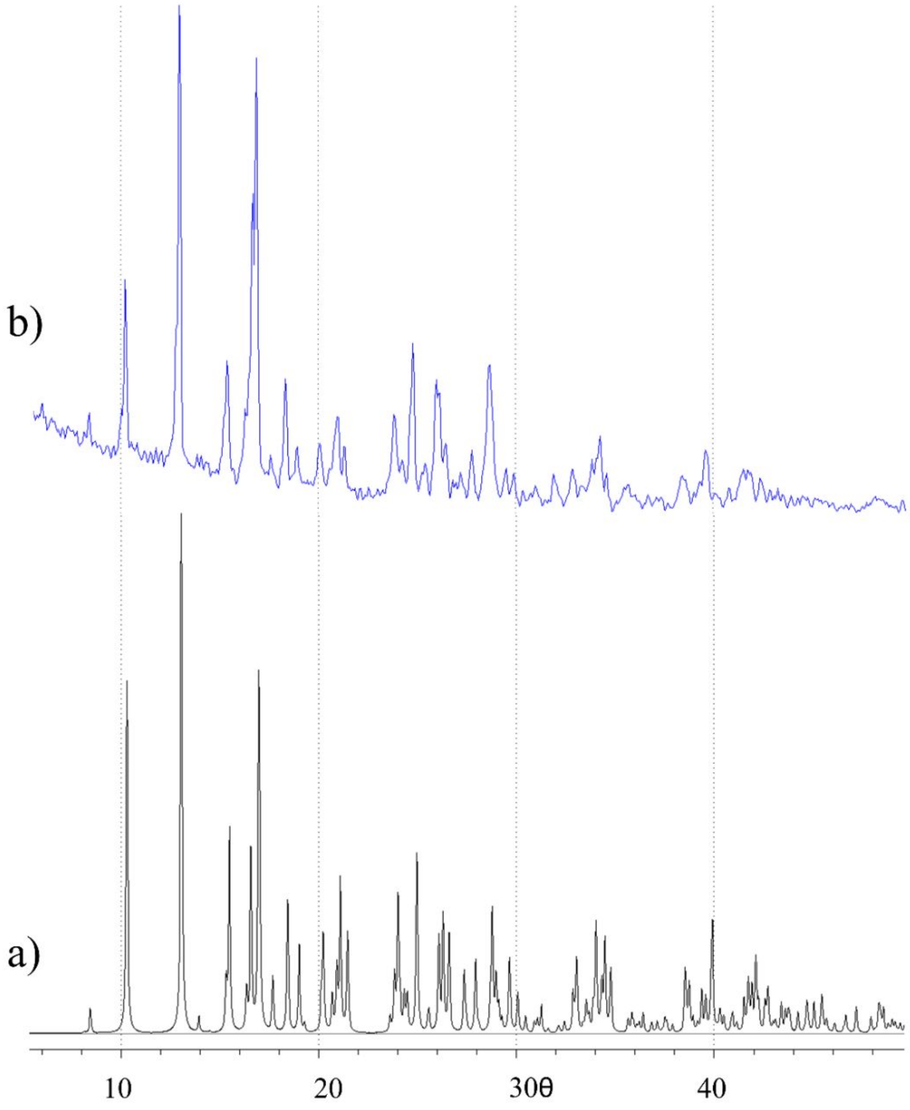

Figure S1. PXRD patterns of compound **1**: (a) calculated from the single crystal structure, (b) bulk microcrystalline product.

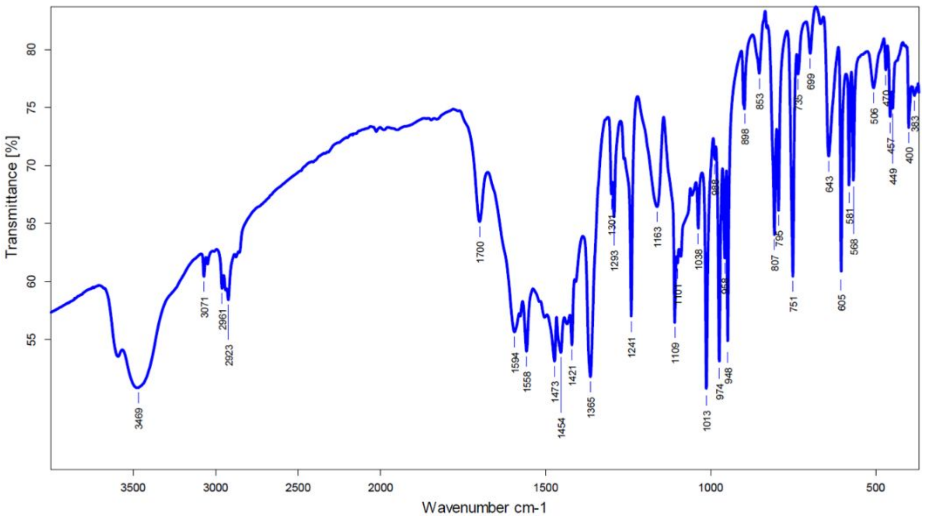

Figure S2. IR spectrum of **1**.

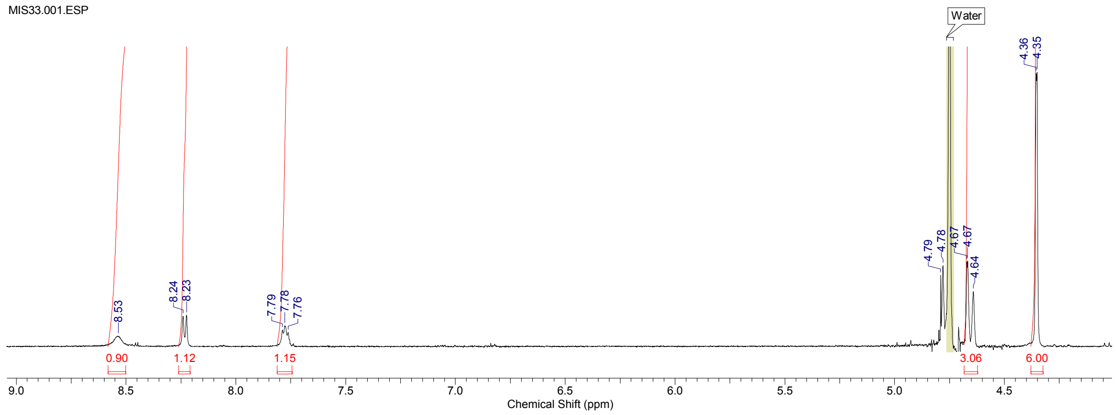

Figure S3. ^{1}H NMR spectrum of **1** (D_2O, r.t., Bruker 500 AMX).

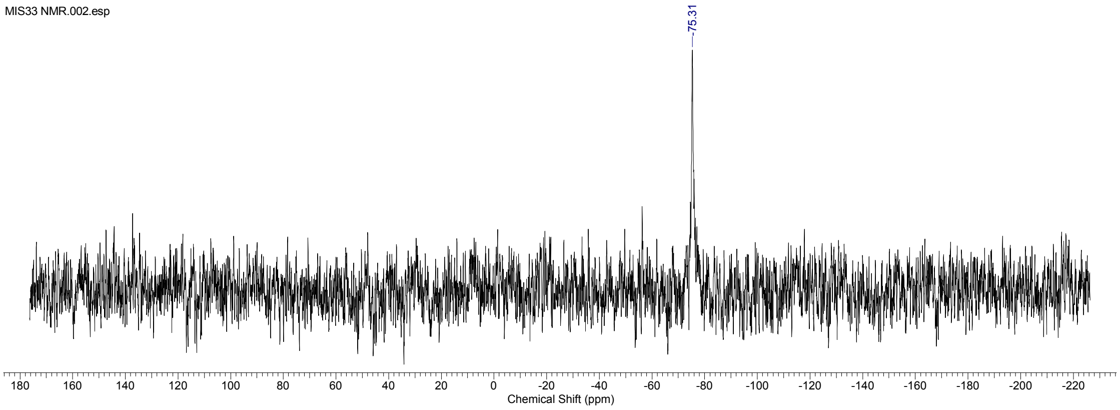

Figure S4. $^{31}P\{^{1}H\}$ NMR spectrum of **1** (D_2O, r.t., Bruker 500 AMX).

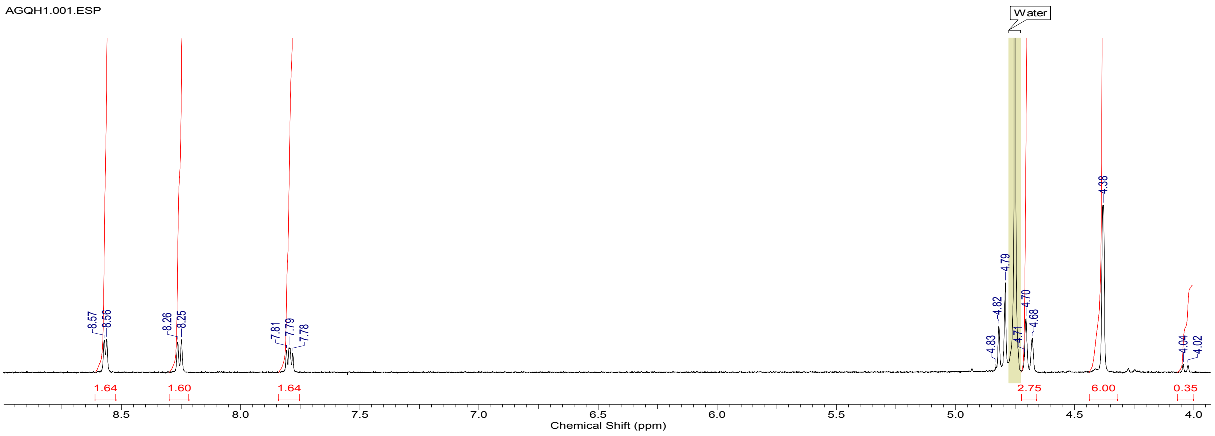

Figure S5. ^{1}H NMR spectrum of **1** (D_2O, r.t., Bruker 600 AMX, after one week).

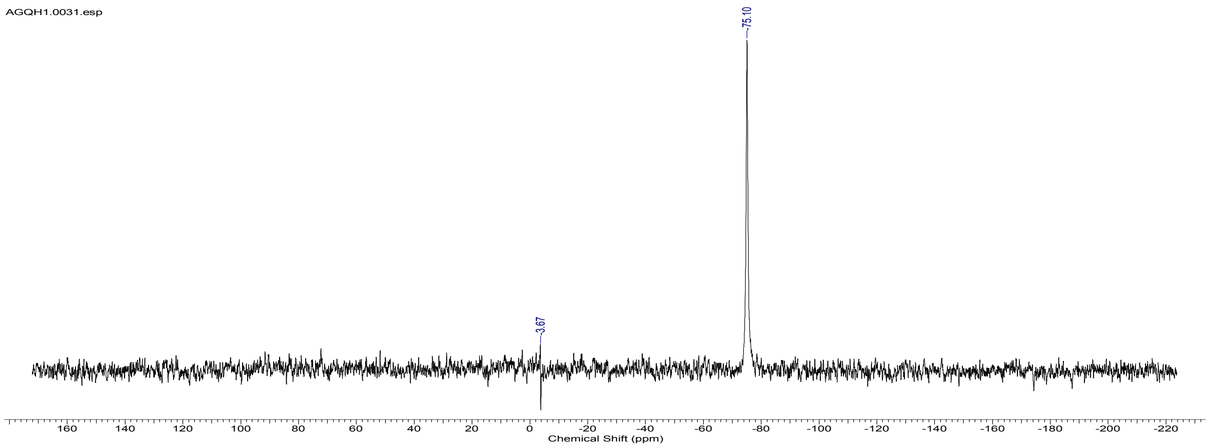

Figure S6. $^{31}P\{^{1}H\}$ NMR spectrum of **1** (D_2O, r.t., Bruker 600 AMX, after one week).

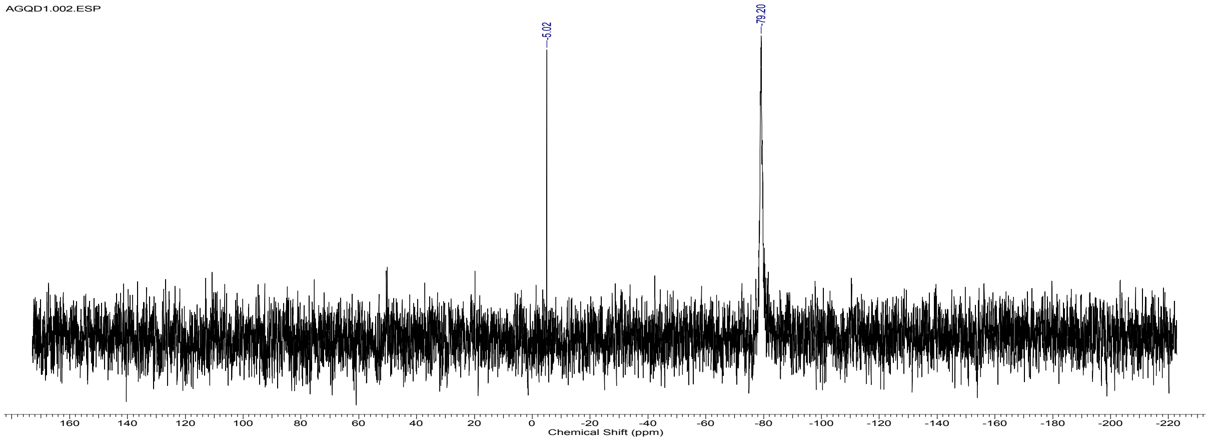

Figure S7. $^{31}P\{^{1}H\}$ NMR spectrum of **1** (DMSO-d_6, r.t., Bruker 600 AMX, after one day).

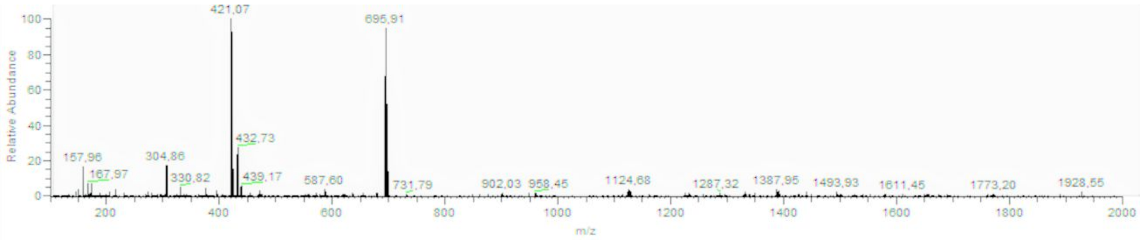

Figure S8. ESI-MS(+) spectrum of **1**.

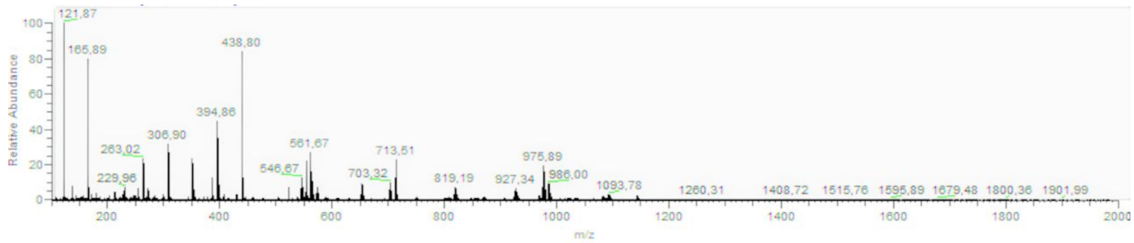

Figure S9. ESI-MS(-) spectrum of **1**.

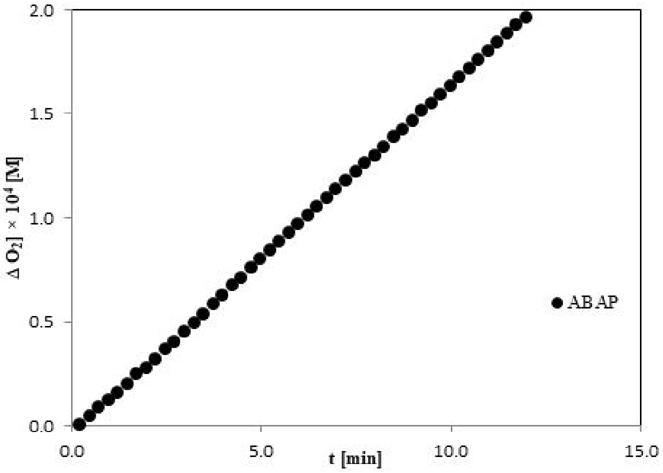

Figure S10. Oxygen uptake for autoxidation of micellar system initiated with ABAP at 37 °C, pH 7.0 Autoxidation without any additive. Final concentration: LinMe 2.74 mM, Triton X-100 8 mM).

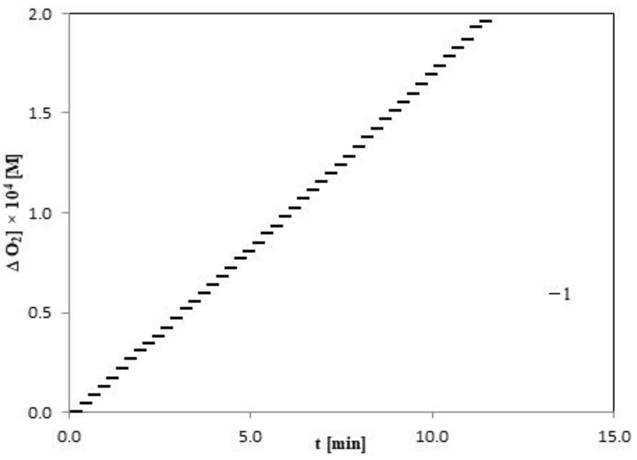

Figure S11. Oxygen uptake for autoxidation of micellar system initiated with ABAP at 37 °C, pH 7.0. Autoxidation in the presence of **1** (1 µM). Final concentration: LinMe 2.74 mM, Triton X-100 8 mM).

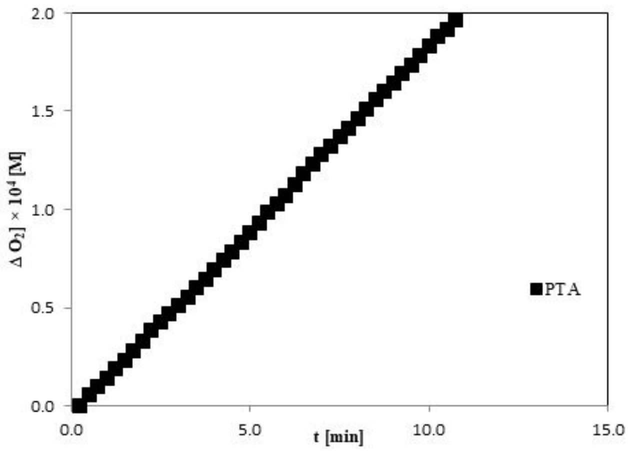

Figure S12. Oxygen uptake for autoxidation of micellar system initiated with ABAP at 37 °C, pH 7.0. Autoxidation in the presence of PTA (1 µM). Final concentration: LinMe 2.74 mM, Triton X-100 8 mM).

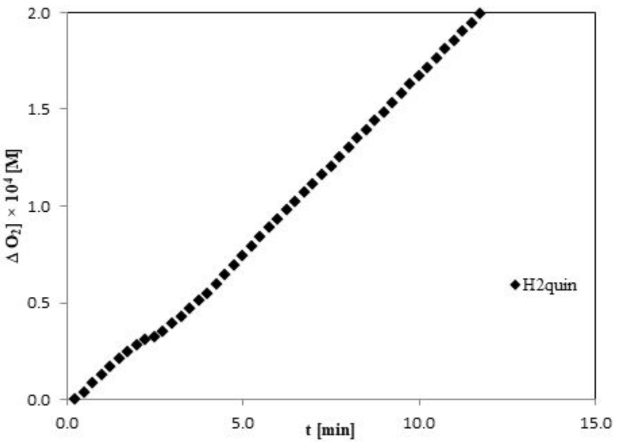

Figure S13. Oxygen uptake for autoxidation of micellar system initiated with ABAP at 37 °C, pH 7.0. Autoxidation in the presence of H_2quin (1 µM). Final concentration: LinMe 2.74 mM, Triton X-100 8 mM).

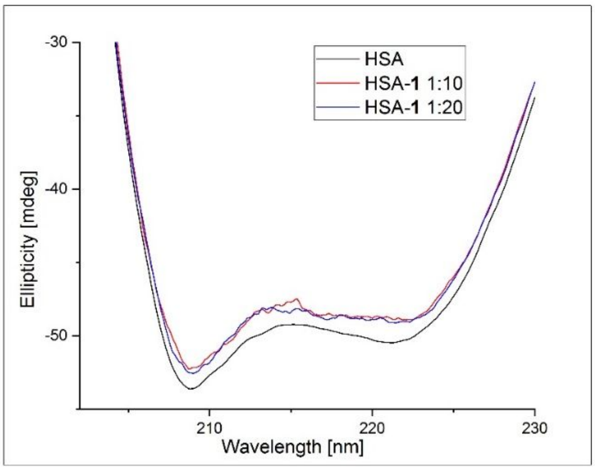

Figure S14. CD spectra of the HSA-**1** systems. [HSA] = 4 µM, 310 K, 24 h.

Table S1. Binding and thermodynamic parameters of the interaction of HSA with **1**.

<table>
<tr><th>T [K]</th><th>K_{sv} [M^{-1}]</th><th>K_q [$M^{-1}s^{-1}$]</th><th>K_a [M^{-1}]</th><th>n</th><th>ΔH [kJ/mol]</th><th>ΔS [J/mol]</th><th>ΔG [kJ/mol]</th></tr>
<tr><td>298</td><td>3.14×10^3</td><td>3.14×10^{11}</td><td>1.19×10^4</td><td>1.08</td><td rowspan="2">−124.17</td><td rowspan="2">−338.44</td><td>−23.27</td></tr>
<tr><td>310</td><td>2.06×10^4</td><td>2.06×10^{12}</td><td>1.73×10^3</td><td>0.891</td><td>−19.20</td></tr>
</table>
